# Correction to: Community mobilisation approaches to preventing and reducing adolescent multiple risk behaviour: a realist review protocol

**DOI:** 10.1186/s13643-021-01720-7

**Published:** 2021-06-07

**Authors:** Laura Tinner, Deborah Caldwell, Rona Campbell

**Affiliations:** Population Health Sciences, Bristol Medical School, Canynge Hall, University of Bristol, Bristol, BS8 2PL UK

**Correction to: Syst Rev 10:147, (2021)**

https://doi.org/10.1186/s13643-021-01696-4

In the original publication of this article [1], the authors’ names, Laura Tinner, Deborah Caldwell and Rona Campbell, were incorrectly captured. The given names and family names were interchanged. The original article has been corrected.
