# Supplementary material for: Community mobilisation approaches to preventing and reducing adolescent multiple risk behaviour: a realist review protocol
Source: Syst Rev. 2021 May 12;10:147. doi: 10.1186/s13643-021-01696-4 (PMC8117311; doi:10.1186/s13643-021-01696-4)
Supplement: Supplementary file 2 — Additional file 2. Search StrategyR1. [file 13643_2021_1696_MOESM2_ESM.docx]

Medline search strategy

Limits: 1946 – 2021, no other limits set.

1. ("Health risk behavio?r*" or "multiple risk behavio?r*" or "high risk behavio?r*" or "multiple risk factor*" or "behavio?r* risk factor*").mp.

2. Dangerous Behavior/

3. Risk-Taking/

4. 1 or 2 or 3

5. "Tobacco Use Disorder"/

6. Smoking/

7. smoking.mp.

8. ((tobacco or cigarette* or nicotine) adj3 (addict* or use* or usage or using or intake or consum*)).mp.

9. 5 or 6 or 7 or 8

10. exp Drinking Behavior/

11. exp Alcohol-Related Disorders/

12. ((alcohol* or ethanol or beer or cider or wine or spirit* or alcopop*) adj3 (use* or usage* or using or intake or consum* or drink* or misus* or abus*)).mp.

13. ((alcohol* or drink* or ethanol) adj3 (excess* or binge* or binging or intoxicat* or poison* or risk* or depend*)).mp.

14. 10 or 11 or 12 or 13

15. cannabis/ or exp street drugs/ or marijuana smoking/

16. Drug-Seeking Behavior/

17. Substance-Related Disorders/

18. ((marijuana or cannabis or recreational drug* or class c or white widow*) adj2 (abus* or use* or using or usage or misus*or smok* or addict* or depend*)).mp.

19. substance abuse, intravenous/

20. (class c adj2 (abus* or addict* or depend* or misus* or use* or usage or using)).mp.

21. (substance* adj2 (abus* or addict* or depend* or inject* or intravenous or misus* or use* or usage or using)).mp.

22. ((Class a or class b or drug* or cocaine or ecstasy or mdma or glue or gas or aerosol* or solvent* or magic mushroom* or crack or ketamine or heroin or morphine or narcotic* or opiat* or opioid* or popper* or lsd or methamphetamine* or amphetamine*) adj2 (abus* or addict* or depend* or inhal* or misus* or sniff* or use* or using or usage)).mp.

23. (inhal?nt* adj2 (abus* or addict* or depend* or misus* or sniff* or use* or using or usage)).mp.

24. 15 or 16 or 17 or 18 or 19 or 20 or 21 or 22 or 23

25. (gambl* or betting).mp.

26. Safe sex/ or unsafe sex/ or sexual behavior/ or sexual abstinence/ or exp contraceptive devices/ or contraceptive agents/ or exp contraception/ or exp reproductive behavior/ or sexual partners/

27. (risky sex* or unsafe sex* or safe* sex* or sexual intercourse or reproductive behavio?r* or sexual behavio?r* or sexual health).mp.

28. (contracept* or condom? or morning after pill*).mp.

29. 26 or 27 or 28

30. exp crime/ or juvenile delinquency/ or social behaviour disorders/

31. violence/ or exp aggression/ or wounds, stab/

32. (delinquen* or offen* or reoffend* or violen* or theft* or robbery or burglary* or steal* or criminal damage or joyrid* or joy-rid* or assault* or (sell* adj drug*) or devian* or anti-social behavio?r* or antisocial behavio?r* or graffiti or racist abuse or index crime* or (breaking adj entering) or strong-arming or pan-handling or panhandling or disorderly conduct or prostitut* or (carry* adj2 weapon*) or (buy* adj2 stolen) or criminal behavio?r* or (noisy adj1 rude) or (nuisance* adj2 neighbour*) or fight* or stab? or stabbing or stabbed or stabwound* or wound* or aggress* or weapon* or knife* or knives or gun* or firearm* or murder*).mp.

33. ((youth* or street or criminal* or adolescen* or juvenile* or teen*) adj2 gang?).mp.

34. ((adolescen* or youth* or juvenile* or delinquen* or teen* or gang? or school* or college* or sixth form*) adj2 (crim* or offen* or violen* or fight*)).mp.

35. 30 or 31 or 32 or 33 or 34

36. Head protective devices/ or exp accident prevention/ or risk reduction behavior/

37. ((bicyc* or cycl* or bik* or motorbike* or motorcycl*) adj2 ((helmet* or protect* or risk reduc* or head gear or head protection) adj3 (lack* or no? or without or absen* or wear*))).mp.

38. Seat Belts/

39. ((Use* or using or usage or wear* or wore) adj2 (seat-belt* or seatbelt* or safety belt*)).mp.

40. (injur* adj2 behav*).mp.

41. ((alcohol* or intoxica* or dr?nk*) adj2 (driv* or vehicle* or motor* or car? or van? or automobile* or auto mobile*)).mp.

42. 36 or 37 or 38 or 39 or 40 or 41

43. suicide, attempted/

44. ((suicid* or harm* or injur* or hurt*) adj2 (gesture* or behavio?r*)).mp.

45. (para-suicid* or parasuicid* or attempt* suicid* or suicid* attempt*).mp.

46. ((non fatal or nonfatal) adj2 (suicid* or harm*)).mp.

47. Self-injurious behavior/ or self mutilation/

48. ((injur* or mutil* or harm* or wound* or hurt*) adj2 (self or themsel* or yoursel*)).mp.

49. dsh.tw. or selfinflict*.mp. or self inflict*.mp. or selfinjur*.mp. or selfharm*.mp. or selfmutilat*.mp. or self destructive behavio?r*.mp.

50. 43 or 44 or 45 or 46 or 47 or 48 or 49

51. exp diet/ or hyperphagia/ or dietary fats/

52. ((calori* or fat? or fatty or fizz* or soft* or carbonated* or sweetened or salt* or sugar* or fruit* or veg? or vegetable* or fibre* or fiber* or 5-a-day or five a day or go for 2&5) adj2 (intake or food* or diet* or consum* or meal* or eat* or nutrition or drink* or snack*)).mp.

53. ((poor or over* or unhealthy or health*) adj3 (nutrition or diet* or eat* or meal* or food* or snack* or drink*)).mp.

54. 51 or 52 or 53

55. exp exercise/ or physical exertion/ or physical fitness/ or physical endurance/ or fitness/

56. Baseball/ or basketball/ or bicycling/ or boxing/ or dancing/ or football/ or gardening/ or golf/ or gymnastics/ or hockey/ or exp martial arts/ or mountaineering/ or exp racquet sports/ or exp running/ or skating/ or snow sports/ or soccer/ or exp swimming/ or volleyball/ or walking/ or weight lifting/ or wrestling/

57. (Physical activity or fitness or physical* fit* or physical exert* or exercise or aerobic activit* or sport* or aerobic capacity or active lifestyle* or outdoor activit* or gym* or mvpa).mp.

58. ((fitness or leisure) adj2 (class* or regime* or program* or centre* or center*)).mp.

59. ((fit* or sport* or activ* or exercise or physical exer*) adj3 (lack* or low or no or absen*)).mp.

60. 55 or 56 or 57 or 58 or 59

61. Sedentary lifestyle/ or Video games/

62. gaming.mp.

63. ((view* or watch* or play* or game* or gaming or use* or using or usage) adj2 (television or tv or video* or dvd* or screen or comput* or laptop* or media)).mp.

64. ((screen or sedentary or view*) adj2 (time or hour* or minute*)).mp.

65. ((inactiv* or seden* or indoor*) adj3 (lifestyle* or activit*)).mp.

66. 61 or 62 or 63 or 64 or 65

67. (4 and 9) or (4 and 14) or (4 and 24) or (4 and 25) or (4 and 29) or (4 and 35) or (4 and 42) or (4 and 50) or (4 and 54) or (4 and 60) or (4 and 66) or (9 and 14) or (9 and 24) or (9 and 25) or (9 and 29) or (9 and 35) or (9 and 42) or (9 and 50) or (9 and 54) or (9 and 60) or (9 and 66) or (14 and 24) or (14 and 25) or (14 and 29) or (14 and 35) or (14 and 42) or (14 and 50) or (14 and 54) or (14 and 60) or (14 and 66) or (24 and 25) or (24 and 29) or (24 and 35) or (24 and 42) or (24 and 50) or (24 and 54) or (24 and 60) or (24 and 66) or (25 and 29) or (25 and 35) or (25 and 42) or (25 and 50) or (25 and 54) or (25 and 60) or (25 and 66) or (29 and 35) or (29 and 42) or (29 and 50) or (29 and 54) or (29 and 60) or (29 and 66) or (35 and 42) or (35 and 50) or (35 and 54) or (35 and 60) or (35 and 66) or (42 and 50) or (42 and 54) or (42 and 60) or (42 and 66) or (50 and 54) or (50 and 60) or (50 and 66)

68. Community Networks/

69. Community Participation/

70. (Community mobilization or community networks or community groups or Community coalition or community mobilisation or community empowerment).mp. [mp=title, abstract, original title, name of substance word, subject heading word, floating sub-heading word, keyword heading word, organism supplementary concept word, protocol supplementary concept word, rare disease supplementary concept word, unique identifier, synonyms]

71. 68 or 69 or 70

72. (Adolescen* or teen* or young person or young people or youth* or hooligan or young adult* or early adult* or juvenile* or minor? or emerging adult* or girl or boy or apprentice* or FE college* or young m#n or young wom#n or young male* or young female* or under 18* or sixth-form* or secondary education or tertiary education or higher education or further education).mp.

73. child/ or adolescent/

74. 72 or 73

75. 67 and 71 and 74
